# Metabolic Dysfunction-Associated Steatotic Liver Disease in Chronic Hepatitis C Virus Infection: From Basics to Clinical and Nutritional Management

**DOI:** 10.3390/clinpract14060200

**Published:** 2024-11-24

**Authors:** Karina Gonzalez-Aldaco, Luis A. Torres-Reyes, Claudia Ojeda-Granados, Leonardo Leal-Mercado, Sonia Roman, Arturo Panduro

**Affiliations:** 1 Centro Universitario de los Valles, Universidad de Guadalajara, Carretera Guadalajara-Ameca Km. 45.5, Ameca 46600, Jalisco, Mexico; luis.torres9386@academicos.udg.mx; 2 Department of Genomic Medicine in Hepatology, Civil Hospital of Guadalajara, “Fray Antonio Alcalde”, Hospital #278, Col. El Retiro, Guadalajara 44280, Jalisco, Mexico; leonardo.leal3084@alumnos.udg.mx (L.L.-M.); sonia.roman@academicos.udg.mx (S.R.); arturo.panduro@academicos.udg.mx (A.P.); 3 Department of Medical and Surgical Sciences and Advanced Technologies “GF Ingrassia”, University of Catania, 95123 Catania, Italy; claudiaojedagranados@hotmail.com; 4 Health Sciences Center, University of Guadalajara, Guadalajara 44340, Jalisco, Mexico

**Keywords:** obesity, hepatitis C, inflammation, liver damage, diet, hepatocellular carcinoma

## Abstract

Metabolic dysfunction-associated steatotic liver disease (MASLD) is closely associated with obesity and other cardiometabolic risk factors. MASLD has rapidly become the most common cause of liver disease worldwide, currently affecting 38% of the global population. Excess weight causes chronic inflammation and the activation of different pathways involved in liver damage. MASLD can progress from simple steatosis to steatohepatitis, giving way to its inflammatory component, metabolic dysfunction-associated steatohepatitis (MASH), previously recognized as non-alcoholic steatosis hepatitis (NASH). Chronic hepatitis C virus (HCV) infection remains a significant challenge to liver health as it triggers hepatic inflammation, metabolic disruption, and hepatic steatosis. The convergence of MASLD and chronic HCV infection can significantly alter the course of liver disease and accelerate the progression to severe liver damage. Currently, HCV treatment has a high cure rate. However, in patients who achieve a sustained virological response after treatment with direct-acting antivirals, weight gain, and excessive calorie intake may contribute to increased liver steatosis and a higher risk of liver disease progression. Therefore, the effective clinical and nutritional management of HCV patients, both before and after viral eradication, is crucial to reducing the risk of death from hepatocellular carcinoma. Understanding the complex interactions between MASLD and HCV infection is crucial for managing these patients appropriately. Herein, host and viral mechanisms inducing liver damage during the coexistence of MASLD and HCV infection are described, and their therapeutic and dietary management are discussed.

## 1. Introduction

Metabolic dysfunction-associated steatotic liver disease (MASLD) has become the most common chronic liver disease worldwide because of the obesity epidemic. According to recent estimations, MASLD affects nearly one-third of the global population [1]. MASLD commonly progresses from simple steatosis to metabolic dysfunction-associated steatohepatitis (MASH) due to inflammatory components [2]. Several factors, including environmental, metabolic, immune, genetic, and epigenetic factors, can affect the progression of MASLD to severe forms of the disease, such as liver fibrosis, cirrhosis, and hepatocellular carcinoma (HCC) [3]. Hepatitis C (HCV) is a hepatotropic virus that disrupts hepatic metabolism, causing progressive liver damage [4]. Currently, chronic hepatitis C ranks among the leading causes of liver transplantation, and it is estimated that 242,000 people worldwide die each year from complications related to hepatitis C [5]. HCV itself can generate hepatic steatosis through mechanisms dependent on viral genotype. Both MASLD and chronic hepatitis C can independently contribute to liver-related complications, and their coexistence may have additive effects on liver health, especially by accelerating liver damage. In this sense, patients with untreated HCV had a prevalence of MASLD of 55%, spanning from 40 to 86% based on the regionality of the metabolic syndrome and HCV genotype [6]. Furthermore, the occurrence of liver steatosis before and after treatment with direct-acting antiviral agents (DAAs) is related to a lack of an improvement in liver fibrosis and an increased risk of liver cancer [7]. Therefore, the adequate clinical and nutritional management of these patients is essential for preventing deaths due to HCC.

A synergistic interaction between the metabolic components associated with HCV infection and MASLD that may accelerate the development of liver damage and HCC has been documented [8]. Thus, the early diagnosis of MASLD in HCV-infected patients would allow for prompt clinical, therapeutic, and lifestyle interventions to prevent further disease severity. This review examines the mechanisms contributing to liver injury during the convergence of metabolic and viral-induced steatosis, and the role of therapeutic and dietary management is discussed.

## 2. Effect of Chronic Inflammation in MASLD and HCV Infection

**Figure 1:**
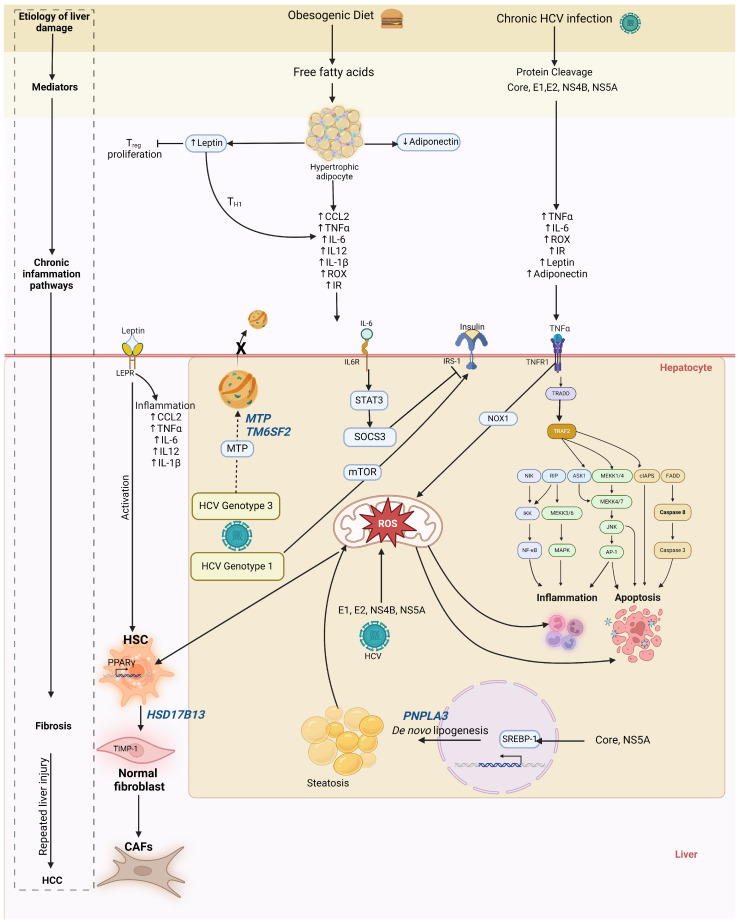
Mechanisms linking MASLD to liver damage during HCV infection. Hypertrophic adipocytes during obesity produce adipokines and other factors that promote intra- and extrahepatic low-grade inflammation. In conjunction with viral proteins, low-grade inflammation accelerates liver damage progression during the coexistence of MASLD+ HCV. The genes involved in MASLD development during HCV infection are highlighted in blue. The arrows with a regular tip represent induction, while the arrows with a blunt tip represent inhibition. The dotted arrow indicates an impaired mechanism effect.

Obesity plays the most crucial role in the onset and progression of simple steatosis and MASH. When the capacity of adipose tissue to store fat is surpassed, hepatocytes begin storing excess lipids, primarily triglycerides. This ectopic fat storage can result in simple steatosis and inflammation. For its part, HCV can induce hepatic inflammation; thus, an intricate hepatic proinflammatory environment prevails during the coexistence of MASLD and chronic HCV infection (Figure 1). Hepatic inflammation is a complex process that protects hepatocytes from injury, favors liver repair, and establishes homeostasis [9]. However, prolonged inflammation leads to hepatocyte death, liver damage, and a decline in liver function [10,11]. Also, chronic inflammation contributes to metabolic disorders and progression from hepatic steatosis to MASH, fibrosis, cirrhosis, and HCC [10].

### 2.1. Adipose-Derived Adipokines Involved in the Onset of Steatosis-Related Liver Damage

Adipose tissue primarily stores fat but can also exert endocrine functions, producing numerous adipokines to regulate metabolic and inflammatory processes [12]. During the progression of excess weight, the adipocyte composition changes by favoring a forward influx of immune cells and overproduction of adipokines [13]. As discussed below, the altered expression pattern of these cytokines is associated with increased liver damage.

#### 2.1.1. Leptin

Leptin is a peptide hormone produced and secreted by adipose tissue. It acts in the hypothalamus to influence food intake, energy expenditure, and fat storage, helping to maintain overall energy homeostasis [14]. Although produced and secreted from adipocytes, leptin acts upon its receptor, LEPR. Dysregulation of leptin production or receptor sensitivity can contribute to metabolic and weight-related disorders [15]. Obese individuals become hyperleptinemic and leptin-resistant due to increased adipogenesis [16]. Circulating leptin in obesity enhances adipocyte and systemic inflammation, up-regulating monocyte chemoattractant protein-1 (MCP-1), also known as CCL2.

Consequently, the infiltration of proinflammatory blood monocyte-derived macrophages and the production of TNF-*α*, IL-6, IL-12, and IL-1β increases [17]. High leptin levels are found in MASLD patients’ liver biopsies [18]. Leptin has shown a potential dual action in MASLD in vitro models. First, leptin was reported as a protective factor for MASLD, safeguarding hepatocyte cells from steatosis and lipotoxicity by preventing the up-regulation of lipogenesis and increasing fatty acid oxidation [19]. In contrast, leptin plays a unique role in developing hepatic fibrosis by activating hepatic stellar cells (HSC) via PPARgamma inhibition and proinflammatory responses (Figure 1) [20,21]. Elevated leptin levels have been observed in patients infected with HCV genotype 1, correlating with an exacerbation of liver steatosis [22,23]. Thus, leptin may play a distinct role in hepatic health depending on the etiology and stage of liver damage.

#### 2.1.2. Adiponectin

Adiponectin is an adipocyte-specific factor contributing to insulin sensitivity, anti-inflammatory responses, and various metabolic processes, including glucose regulation and fatty acid oxidation [24]. Adiponectin mainly binds to its receptor AdipoR2. AdipoR2 activates 5-AMPK and PPAR-α pathways involved in fatty acid oxidation and inhibition of inflammation [25]. Adiponectin also downregulates the hepatic expression of CD36, thereby decreasing the influx of free fatty acids (FFAs) into the liver. Besides metabolic regulation, adiponectin has antifibrotic action in the liver via downregulating AOX-1, TGF-β, and connective tissue growth factor expression [26]. Adiponectin also has anti-inflammatory action in the liver by suppressing TNF-α and other proinflammatory cytokines and inducing anti-inflammatory cytokines, such as IL-10 [27]. Low adiponectin levels have been reported in obese subjects and patients with hepatic steatosis or MASH [28]. Recently, a significant association between elevated serum adiponectin levels and advanced Child-class liver cirrhosis in patients with HCV infection has been reported. ADIPOR1 mRNA were reduced in chronic HCV-infected patients, but not ADIPOR2 levels, suggesting a pattern of adiponectin resistance [29,30]. The beneficial effect of adiponectin is diminished in both MASLD and HCV patients. In the case of chronic hepatitis C, it is necessary to investigate the mechanisms that induce the downregulation of ADIPOR1 expression.

#### 2.1.3. Tumor Necrosis Factor-Alpha (TNFα)

TNFα is a pleiotropic cytokine involved in diverse processes such as cell proliferation, metabolic activation, and inflammatory response [31]. The ligand of TNFα, the ligand-bound TNR receptor 1 (TNFR1), induces the production of reactive oxygen species (ROS) via the NOX1, thus coordinating the proinflammatory response (Figure 1) [32]. It has been reported that TNFR1 favors the recruitment of caspase-8, which initiates an apoptotic cascade in the liver [33]. TNFα expression increases insulin resistance (IR), IL-6, IL-1, and early MASH in diet-induced non-obese MASLD mice [34]. High levels of TNFα have been found in cirrhotic HCV patients as well as in MASH patients [35,36]. Thus, both etiologies may potentiate liver damage via TNFα activity.

#### 2.1.4. Interleukin-6 (IL-6)

IL-6 is a pleiotropic cytokine expressed in different tissues. However, white adipose tissue is responsible for up to 35% of human IL-6 production [37]. IL-6 expression is highly correlated with obesity, contributing to a low grade of inflammation in this condition. IL-6 has differential functions according to tissue type. In the liver, it induces the acute phase response and infection defense, but aberrant activation of the IL-6 pathway can trigger hepatocyte apoptosis and mediate immune liver damage [38]. Also, IL-6 impairs insulin action through the STAT3-SOCS-3 pathway (Figure 1) [39,40]. High levels of IL-6 are reported in MASLD, MASH, and chronic HCV patients. However, the highest levels are found in MASLD patients, rather than in HCV patients. IL-6 activation results in the excessive stimulation of the IL-6/STAT3 signaling pathway in HCC cells. This process can increase the expression of the tissue inhibitor of metalloproteinases-1 (TIMP-1), driving the conversion of normal liver fibroblasts into carcinoma-associated fibroblasts (CAFs), thereby contributing to liver carcinogenesis [41]. Hence, IL-6 could be the key to rapidly conducting a dual MASLD + HCV state toward HCC development.

## 3. Immunometabolic Dysregulation Enhances Liver Damage in MASLD and Chronic HCV

Low-grade inflammation in obesity is characterized by metabolic dysfunction that increases the risk for cardiovascular disease, cancer, and other life-threatening conditions, including liver disease [42,43]. The lipotoxic microenvironment in MASLD increases hepatic oxidative stress, leading to T-cell recruitment, persistent proinflammatory response, and subsequent fibrosis and MASH development [43]. On the other hand, HCV is a metabolic regulator virus that, by itself, can modulate liver metabolism with hepatic insulin resistance and subsequent oxidative damage. Thus, the overlap of these two etiologies can exacerbate liver damage (Figure 1).

### Insulin Resistance and Oxidative Stress

During weight gain, decreased glucose uptake by insulin-resistant skeletal muscle leads to compensatory hyperinsulinemia. Proinflammatory cytokines such as TNF*α* and IL-6 produced in hypertrophic adipocytes cause IR as they can stimulate the signaling pathways of the c-Jun amino-terminal kinase (JNK) and the IκB kinase-β (IKK-β)/nuclear factor-κB (NFκB). Once activated, JNK and NFκB phosphorylate the serine kinase insulin receptor substrate-1 (IRS1) and insulin receptor substrate-2 (IRS-2) that block insulin signaling, resulting in the incidence of IR [44]. In parallel, HCV can trigger hepatic and extrahepatic IR via core-protein-promoting IRS-1 degradation in viral genotype 3, while genotype 1 activates the mTOR signaling, reducing IRS1/2 signaling (Figure 1) [45,46]. The presence of IR has been associated with liver fibrosis in chronic HCV and MASLD patients [47]. Hyperinsulinemia and increased levels of insulin growth factors have been shown to promote cell proliferation in chronic inflammatory conditions, such as MASH and chronic HCV infection [48].

On the other hand, ROS are regular products of the mitochondrial respiratory chain. They constitute a highly reactive species produced in membranes and cytosolic and reticular components and are involved in TNFR1 activation [49]. ROS participates in intracellular liver damage, mitochondrial dysfunction, and cell death. Direct measurements in liver tissue from chronic HCV patients revealed an increase in ROS concentrations by two to five orders of magnitude [50]. One study demonstrated that HCV can potentially cause ROS production via the core, E1, E2, NS4B, and NS5A, the core protein’s most potent regulator [51]. They increased oxidative stress in vivo in a large cohort of subjects with MASLD. High oxidative stress is a well-established cause of liver injury due to indiscriminate oxidative biomolecular damage and dysregulated redox signaling [52]. Thus, HCV patients who have excess weight are expected to accelerate the progression of liver damage.

## 4. Genetic Variants Linked to the Development of MASLD in Hepatitis C Virus Infection

Susceptibility toward MASLD development in chronic HCV patients depends on the host’s genetic background. Genome-wide association studies revealed that many single variants confer susceptibility to steatosis by inducing hepatic fat accumulation [53]. One of the most widely described variants associated with MASLD is the non-synonymous isoleucine-to-methionine substitution at position 148 in the *PNPLA3* gene (rs738409). This single nucleotide polymorphism (SNP) leads to the reduced enzyme activity of triglyceride lipase and reduced retinyl ester hydrolysis, resulting in hepatic triglyceride accumulation and hepatic stellate cell activation and fibrogenesis [54]. Chronic HCV patients who carry the GG genotype have a 4.33-fold increased risk of developing hepatic steatosis and a 2.99-fold increased risk of severe fibrosis compared to carriers of other genotypes [55]. Also, the *PNPLA3* rs738409 and the *HSD17B13* rs72613567TA variants have been associated with more severe liver disease, from mild fibrosis to significant fibrosis, cirrhosis, and HCC in chronic HCV infection [56]. Higher liver triglyceride content is also found in patients with the missense mutation E167K variant (rs58542926) in the *TM6SF2* gene, which decreases hepatic lipid secretion [57]. The *TM6SF2* E167K variant independently predicts steatosis in chronic HCV patients [58]. MTP is a protein involved in the hepatic lipid release by transferring lipids from the endoplasmic reticulum to the nascent apolipoprotein B and very low-density lipoprotein. The *MTP* rs1800591 variant confers a 6.72-fold increased risk of hepatic steatosis in chronic HCV genotype 3 patients [59]. These genetic variants are found in critical enzymes involved in lipid metabolism and can promote liver steatosis, resulting in the onset of liver damage (Figure 1).

## 5. Effect of HCV Genotypes on MASLD

HCV is a genetically heterogeneous virus with one to eight genotypes and over 50 subgenotypes [60]. Each genotype exerts a distinct influence on metabolism during infection, and specific genotypes may favor liver steatosis on their own or in the presence of obesity (Table 1). The development of steatosis in patients with chronic HCV infection ranges between 40–86%, with an average of 55% in all genotypes. The prevalence of liver steatosis in chronic HCV patients is higher than in the general adult population (55% compared to 20–30%) [6]. Genotype 3, also called the “steatogenic genotype”, is associated with a hepatic steatosis prevalence of up to 86% [61]. Patients infected with genotype 3 show more frequent and more severe steatosis with accelerated progression to liver damage and HCC than other genotypes, even without the presence of IR, obesity, or other metabolic risk factors related to MASLD. Genotype 3-induced liver steatosis is proportional to viral load and is resolved after successful viral treatment, indicating a direct cytopathic effect. MASLD related to genotype 3 involves the core protein inhibiting MTP, decreasing hepatic lipid export and triglyceride accumulation [62]. Genotype 3a activates p-Akt, increasing fatty acid synthesis via SREBP-1 and decreasing lipolysis by inhibiting PPARα [63,64].

On the other hand, genotypes 1, 2, and 4 promote steatosis primarily associated with pre-existing host metabolic risk factors, such as IR and visceral obesity [65]. The activation of proinflammatory pathways in obesity and IR is related to the emergence of steatosis in these patients. Also, increased free radical levels (MDA > 250 nmol/dL) have been significantly correlated with liver fibrosis in patients with an HCV genotype 1 [66]. However, no relationship has been found between genotype 1-viral load and the achievement of a sustained virologic response (SVR) or the extent of liver steatosis [67].

## 6. Treatment Considerations in Patients with MASLD and HCV Infection

The goal of HCV treatment is to reduce the occurrence of end-stage liver disease and its complications. Treatment success is assessed by SVR, which is defined as undetectable HCV RNA in the blood several months after treatment. Initially, HCV was treated with interferon (IFN)-based regimens. Nonetheless, the presence of steatosis and metabolic syndrome was recognized as a negative factor in antiviral IFN-based therapy [68,69]. Efforts to eliminate HCV have activated an ongoing global campaign to diminish the incidence and mortality caused by this virus [70].

**Figure 2:**
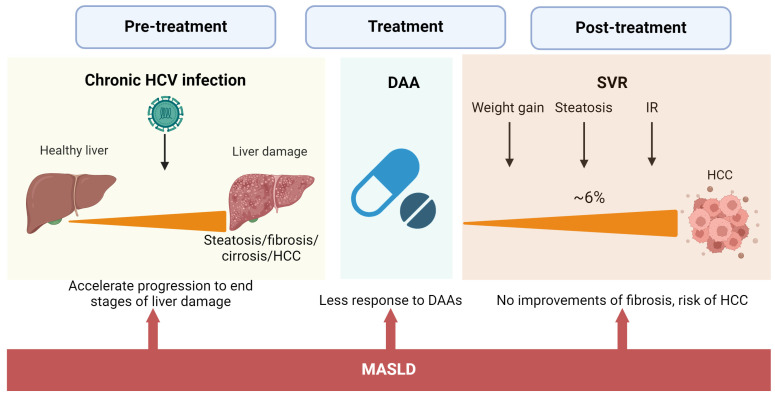
Impact of MASLD on the natural history of HCV infection and after DAA treatment. The concurrence of MASLD and chronic HCV affects the natural history of HCV infection, accelerating the occurrence of advanced stages of liver damage. High BMI during and after treatment is associated with less SVR and no improvements in fibrosis grade. DAA effects on liver steatosis and metabolism significantly increase the risk of HCC even after SVR.

Today, pharmacological treatment with DAAs against HCV has reached a high cure rate among all HCV genotypes [71]. However, some questions remain to be solved because post-treated patients and those who may be unaware of their conditions are at risk of HCV-induced HCC. (Figure 2).

These patients with metabolic-associated steatotic conditions need medical and nutritional intervention. Eradication of HCV by DAAs is associated with weight gain, liver steatosis, and no improvement in IR [7,72,73]. A recent study of 1280 elderly patients with HCV eradication by DAAs and no history of HCC demonstrated that 25.8% of the patients developed MASLD at 24 weeks of SVR. In turn, MASLD at 24 weeks of SVR conferred a 3.04-fold increased risk of developing HCC. In the end, 6.7% of the patients developed HCC [74]. Thus, patients with dual etiology of liver damage are at higher risk for advanced hepatic fibrosis and HCC even after HCV eradication.

Similarly, having obesity before treatment with DAAs is negatively associated with improvement of fibrosis at 1-year follow-up [75]. Although DAAs have reached a high cure rate, 5% of the patients treated will not respond to therapy. A retrospective study involving 10,655 patients treated with DAAs to determine predictive factors associated with nonresponse to treatment found that an elevated pretreatment body mass index (BMI) was associated with nonresponse to DAAs [76]. Hence, managing MASLD before and after DAA treatment is vital to prevent the progression of the disease and prevent death by HCC.

The treatment of MASLD primarily focuses on modifying risk factors through therapies such as insulin sensitizers, antioxidants, weight reduction, physical activity, and diet [77]. Although there is little evidence regarding weight management before DAA treatment, a study reported that weight loss of >0.5 BMI before IFN + ribavirin therapy was associated with higher SVR [78]. Metformin is a primary insulin sensitizer in clinical practice. An in vitro study demonstrated that metformin inhibits HCV replication, activating the type I IFN antiviral signaling pathway via activation of AMPK and decreasing core protein expression [79]. Metformin also reduces the risk of HCC incidence after SVR among those with diabetes and chronic HCV [80]. When supplemented with metformin, statins lower serum cholesterol, reducing the risk for HCC in chronic patients who failed antiviral therapy [81]. Vitamin E is an antioxidant agent that is beneficial for treating non-diabetic MASLD patients [82]. Vitamin E supplementation of 400 IU twice daily for 12 weeks decreased serum alanine aminotransferase levels in patients infected with HCV genotype 3 [83]. Recently, resmetirom, a liver-targeted selective thyroid hormone receptor-β (THR-β) agonist, became the first FDA-approved drug for treating non-cirrhotic MASH patients. Resmetirom has been shown to improve cholesterol and triglyceride levels and reduce liver fat in MASH patients. However, its potential effects on HCV patients remain unexplored [84,85]. This evidence implies that MASLD management when HCV coexists may benefit both etiologies of liver damage. More studies are needed to investigate the effect of MASLD therapy in patients with both conditions.

## 7. Dietary Considerations in Patients with MASLD and Chronic HCV Infection

Management strategies for MASLD that focus on lifestyle modifications are essential for liver-diseased patients. Dietary interventions can improve inflammation, oxidative stress, IR, and BMI and reduce liver damage due to MASLD [86,87,88]. Furthermore, it has been documented that dietary interventions are a valuable resource that should be tailored by region based on genetic and environmental (cultural) differences among populations [89]. For example, epidemiological studies performed on US Hispanics of Mexican descent showed higher rates of obesity, diabetes, and MASLD [90], as well as higher rates of genetic susceptibility compared to other ethnic groups [1,91]. Studies in Mexican subpopulations have reported anthropometric and biochemical alterations in young obese patients with MASLD who consumed a hepatopathogenic diet [92]. However, it has also been documented that adherence to a traditional Mexican or genome-based diet in this population, i.e., consumption of a diet integrating Mexican staple foods such as maize products, legumes, pumpkin, zucchini, prickly pears, chia seeds, amaranth, tomato, squash, and chili with anti-inflammatory, antioxidant, anti-fibrogenic, and insulin-sensitizing properties, could decrease the risk of MASLD-related conditions [93,94].

Additionally, several foods and their components have shown beneficial effects for MASLD, such as preventing inflammation, decreasing cardiometabolic risk factors, and reducing liver fibrosis [95]. The availability and quantity of each micronutrient may depend on each population’s diet and food culture. As shown in Table 2, dietary recommendations for MASLD patients based on nutrients or functional components with beneficial effects on liver steatosis can significantly help with the clinical management of the disease.

The HCV lifecycle is closely related to hepatic lipid metabolism. Lipid droplets are involved in the HCV replication, assembly, and release stages. While saturated fatty acids (SFA) are required for successful HCV replication, polyunsaturated fatty acids (PUFAs) inhibit in vitro HCV replication [114,115]. A study revealed that an SFA-rich diet in HCV core protein transgenic mice increases liver steatosis, lipogenesis, inflammation, and the presence of liver tumors [115]. This study suggests that excessive intake of SFA-rich foods should be avoided in HCV-infected patients to prevent liver cancer. However, this finding might apply to MASLD patients or MASLD + HCV patients as the study reproduces steatosis-derived liver tumorigenesis without significant fibrosis. Another in vitro study demonstrated that arachidonic (AA), docosahexaenoic (DHA), and eicosapentaenoic acids (EPA) inhibit HCV replication by suppressing the expression of genes involved in lipogenesis [96,97]. However, the exact mechanism underlying these effects is unclear.

**Figure 3:**
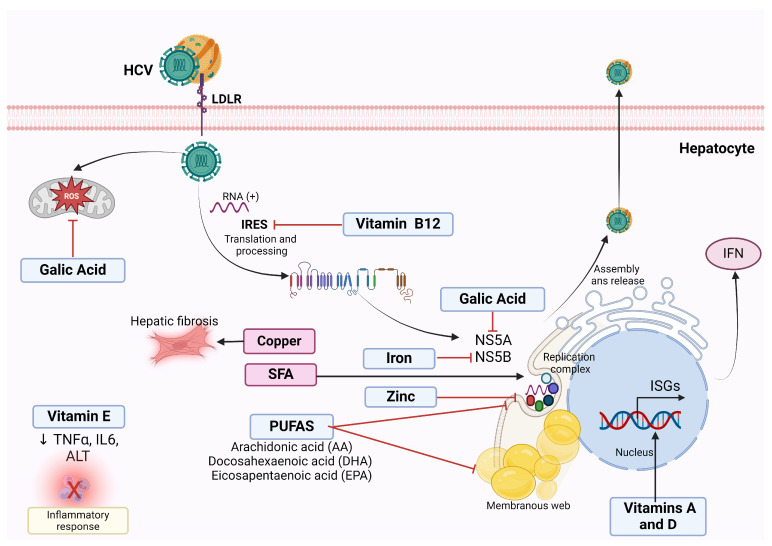
Antiviral effect of nutrients against HCV infection. Polyunsaturated fatty acids (PUFAs) inhibit the formation of the membranous web necessary for successful HCV replication in vitro, thus reducing viral RNA replication and virus production. Gallic acid downregulates the expression of NS5A HCV protein required for HCV replication and decreases the ROS derivates from HCV infection in vitro. Vitamin B12 inhibits HCV internal ribosome entry site (IRES), essential for HCV translation, thus limiting HCV persistence in vitro. Vitamin E reduces TNFα and IL-6, suggesting an anti-inflammatory effect. Vitamins A and D induce the transcription of type-1 IFNs, enhancing the effect of IFN on HCV. Iron Inhibits NS5B polymerase activity in vitro. Zinc reduces HCV replication in vitro. On the contrary, saturated fatty acids (SFA) are necessary for HCV replication. Hepatic copper increases hepatic fibrosis and correlates positively with type IV collagen.

Similarly, several micronutrients have demonstrated anti-HCV replication in vitro (Figure 3, Table 2), but their effect in vivo is scarcely known [116]. BMI and dietary patterns of patients with an active HCV infection can influence the disease outcome [117,118]. A recent study of chronic HCV-infected patients found that patients with adherence to a fish-rich dietary pattern consisting mainly of fish, seafood, vegetable oils, and PUFAs ≥ 4.9% had lower viral load levels [118].

In this sense, PUFAs can decrease inflammation, ameliorate insulin sensitivity and liver steatosis, and counteract core protein effects [97]. DHA, EPA, and AA supplementation may help prevent or treat MASLD associated with HCV infection. Because of their absence of adverse effects, these treatments might be suitable for adults and children. Dietary intervention with PUFAs in MALSD + HCV patients could benefit both etiologies of liver damage. Furthermore, clinical practice guidelines must consider nutritional recommendations for patients with HCV infection to offer standard recommendations to the public.

## 8. Conclusions

There are numerous similarities between fat accumulation in HCV and MASLD. Besides fatty liver accumulation, HCV resembles MASLD in terms of IR, oxidative stress, metabolic dysfunction, and chronic inflammation. Both MASLD and HCV can independently trigger liver damage. When these two etiologies of liver damage converge, intricated mechanisms involving low-grade inflammation, IR, oxidative stress, and metabolic disturbances, as well as viral proteins, enhance the progression of liver damage. Future perspectives for managing MASLD in HCV patients should emphasize the development of clinical practice guidelines that integrate general and region-specific nutritional recommendations [119]. These guidelines are crucial for providing standardized advice tailored to diverse populations, particularly in regions where achieving HCV eradication remains challenging. Addressing metabolic disturbances must also be a priority, especially in patients with liver steatosis, but also in those without fatty liver before DAA treatment. Mandatory clinical follow-up should be implemented for all HCV patients after viral eradication, considering the risk of liver steatosis and HCC. A comprehensive approach combining clinical, nutritional, and metabolic management is essential for improving long-term outcomes in patients with MASLD and HCV chronic infection. HCC incidence will be an important health issue in HCV patients after virus eradication.

## Figures and Tables

**Table 1:** Disease characteristics of liver steatosis in HCV genotype 3 and non-genotype 3.

	Genotype 3	Non-Genotype 3
Term	Viral steatosis	Metabolic steatosis
Mediator	Core protein	Host metabolic disturbances
Mechanism	↓ MTP, ↓ VLDL, and ↓ lipid secretion	↓ IRS1,↓ PI3K-Akt, ↑ IR, ↓ PPARγ, ↑ CD36, ↑ SREBP
Severity	Accelerate steatosis, rapid progression to fibrosis, and HCC	Lower rate of steatosis, slower progression to fibrosis, and HCC
Correlation with antiviral therapy	Reversible after SVR	No improvement after SVR

MTP: microsomal triglyceride-transfer protein; VLDL: very low-density lipoprotein; IRS1: insulin receptor substrate 1; PI3K-Akt: phosphatidylinositol 3-kinase (PI3K)/Akt; IR: insulin resistance; PPARγ: peroxisome proliferator-activated receptor γ; CD36: scavenger receptor B2; SREBP: sterol regulatory element binding protein; HCC: hepatocellular carcinoma; SVR: sustained virological response; ↑: induction; ↓: reduction.

**Table 2:** Food source and concentration of nutrients with anti-HCV and MASLD activities.

Nutrient	Food Source	Amount *	Effect	Ref.
**Anti-HCV**				
DHA	Fish oil—salmon	18.2 g	HCV inhibition in vitro. Counteract core protein-lipid alterations.	[96,97]
	Fish oil—cod liver	11 g		
	Egg—yolk, dried	0.253 g		
	Fish—carp, raw	0.114 g		
EPA	Fish oil—salmon	13 g		
	Fish oil—cod liver	6.9 g		
	Fish—herring, Pacific, cooked, dry heat	1.24 g		
AA	Fish oil—sardine	1.76 g		
	Egg—yolk, dried	0.978 g		
	Fish oil—cod liver	0.935 g		
	Beef—variety meats, and by-products	0.74 g		
Gallic acid	Chestnut—raw	479.78 mg	↓ HCV expression through its antioxidant capacity	[98]
	Cloves	458.19 mg		
	Oregano—dried (wild marjoram)	5.15 mg		
	Black Tea—infusion	4.63 mg		
	Blackberry—raw	4.67 mg		
Vitamin E	Chili powder	38.1 mg	↓ ALT and favors inflammatory response	[83]
	Sunflower seed kernels—oil roasted	36.3 mg		
	Nuts—almonds, oil roasted, without salt	26.0 mg		
	Oregano—dried	18.3 mg		
Vitamin A	Duck—domesticated, liver, raw	39,900 IU	↑ anti-viral effect of IFN on HCV replication	[99]
	Veal—variety meats, liver, cooked, braised	70,600 IU		
	Pork—fresh, variety meats and by-products	21,600 IU		
	Carrots—cooked, boiled, drained	17,000 IU		
	Broccoli—leaves, raw	16,000 IU		
	Pumpkin with salt	15,600 IU		
Vitamin D3	Fish—carp, raw	24.7 µg	Inhibit HCV replication by modulating IFN signaling	[100]
	Egg—yolk, dried	15.7 µg		
	Egg—whole, dried	9.7 µg		
Vitamin B12	Veal—variety meats and by-products	84.6 µg	Inhibit HCV translation directed by all IRES elements	[101]
	Beef—variety meats and by-products	83.1 µg		
	Duck—domesticated, liver, raw	54.0 µg		
	Pork—fresh, variety meats and by-products	26.0 µg		
Iron	Marjoram dried	82.7 mg	Inactivates HCV NS5B protein and suppresses subgenomic replication	[102]
	Cumin seed	66.4 mg		
	Turmeric—ground	55.0 mg		
	Beef—variety meats, and by-products	44.6 mg		
Zinc	Agave—dried (Southwest)	12.1 mg	Inhibit HCV replication	[103]
	Beef—chuck, short ribs, boneless, cooked	12.1 mg		
	Seeds—sesame flour (ajonjoli)	10.7 mg		
**Anti-MASLD**				
Polyphenols (resveratrol, quercetin, catechin, and cyanidin)	Cocoa—chocolate, dark	Quercetin: 25.0 mg Catechin: 20.50 mg Resveratrol: 0.04 mg	Protect against steatosis, mitochondrial dysfunction, and impaired energy metabolism	[104]
	Mexican oregano—dried	Quercetin: 42.0 mg		
	Beans—common bean	Cyanidin: 1.63 mg		
Epicatechin	Cocoa—chocolate, dark	70.36 mg	↓ response of PPARα and PPARγ in vitro	[105]
	Broad bean pod—raw	37.55 mg		
	Green tea—infusion	7.93 mg		
Vitamin E	Chili powder	38.1 mg	Improvement of serum liver markers, inflammation and histology of MASLD patients	[106]
	Sunflower seed kernels—dried	35.2 mg		
	Almonds	25.6 mg		
	Oregano—dried	18.3 mg		
PUFAs (EPA, DHA)	View anti-HCV section	View anti-HCV section	↓ GGT and liver fat. Beneficial changes in lipid profile of MASLD patients	[107]
Vitamin D	View anti-HCV section	View anti-HCV section	↓ ALT, AST, FBS, LDL-c, miR-21, and miR-122 in MASLD patients	[108]
Curcumin	Turmeric—dried	2213.57 mg	↓ insulin resistance, steatosis in obese mice	[109]
	Curry—powder	285.26 mg		
Chlorogenic Acid	Oregano—dried (wild marjoram)	10.70 mg	Protect against steatosis in HepG2 cells	[110]
	Cumin	16.60 mg		
	Sunflower seed	8.17 mg		
Combination of soy protein, chia oil, curcumin, and nopal	Soy, chia, curcumin, and nopal	NA	Modifies gut microbiota, ↓ hepatic fat and ↑ mitochondrial function	[111]

* Nutrient concentrations are given per 100 g or mL portion. Food sources and concentrations were obtained from U.S. Department of Agriculture Database [112] or the Phenol-Explorer Database [113]. NA: not applicable; Ref.: reference; ↑: increase; ↓: decrease.

## Data Availability

No new data were created or analyzed in this study. Data sharing is not applicable to this article.
